# Update on COVID-19 Therapy in Pediatric Age

**DOI:** 10.3390/ph15121512

**Published:** 2022-12-05

**Authors:** Susanna Esposito, Giovanni Autore, Alberto Argentiero, Greta Ramundo, Serafina Perrone, Nicola Principi

**Affiliations:** 1 Pediatric Clinic, Department of Medicine and Surgery, University of Parma, 43126 Parma, Italy; 2 Neonatoloy Unit, Department of Medicine and Surgery, University of Parma, 43126 Parma, Italy; 3 Università degli Studi di Milano, 20122 Milan, Italy

**Keywords:** antiviral therapy, bebtelovimab, COVID-19, molnupiravir, nirmatrelvir/ritonavir, remdesivir

## Abstract

With the extension of the COVID-19 pandemic, the large use of COVID-19 vaccines among adults and the emergence of SARS-CoV-2 variants means that the epidemiology of COVID-19 in pediatrics, particularly among younger children, has substantially changed. The prevalence of pediatric COVID-19 significantly increased, several severe cases among children were reported, and long-COVID in pediatric age was frequently observed. The main aim of this paper is to discuss which types of treatment are presently available for pediatric patients with COVID-19, which of them are authorized for the first years of life, and which are the most important limitations of COVID-19 therapy in pediatric age. Four different antivirals, remdesivir (RVD), the combination nirmatrelvir plus ritonavir (Paxlovid), molnupiravir (MPV), and the monoclonal antibody bebtelovimab (BEB), are presently approved or authorized for emergency use for COVID-19 treatment by most of the national health authorities, although with limitations according to the clinical relevance of disease and patient’s characteristics. Analyses in the literature show that MPV cannot be used in pediatric age for the risk of adverse events regarding bone growth. The other antivirals can be used, at least in older children, and RDV can be used in all children except in neonates. However, careful research on pharmacokinetic and clinical data specifically collected in neonates and children are urgently needed for the appropriate management of pediatric COVID-19.

## 1. Introduction

Since the declaration of the COVID-19 pandemic, it has been shown that the incidence of SARS-CoV-2 infection in children is significantly lower than in adults and that when infection develops, most infected children remain asymptomatic or develop a mild disease [1,2,3]. With the extension of the pandemic, the large use of COVID-19 vaccines among adults, and the emergence of SARS-CoV-2 variants, the epidemiology of COVID-19 in pediatrics, particularly among younger children, has substantially changed. Compared to adults, the prevalence of pediatric COVID-19 significantly increased [4,5,6,7,8]. Moreover, several severe cases among children were reported. In the period 10 October 2021 to 29 September 2022, the prevalence of pediatric COVID-19 cases on the total number of COVID-19 cases diagnosed in the USA rose from 16.6% to 18.4% [4]. The increase was mainly due to the rise in cases identified in children under 12 years of age: those for whom vaccination programs had been activated in the USA only at the beginning of the evaluation period (children aged 5–12 years) or only a few weeks (children aged < 5 years) before its end [5,6]. Moreover, a greater number of severe COVID-19 cases were described in neonates and young children [7,8]. In the USA, the analysis of weekly hospitalization rates among infants and children aged 0–4 years from 1 March 2020 to 19 February 2022 revealed that, in the last pandemic months, when the Omicron variant was predominant, the peak hospitalization rate was approximately five times higher than the peak of the previous Delta variant period, with the greatest differences when infants aged < 6 months were considered [9]. Moreover, only about one-third of the hospitalized patients had a severe chronic underlying disease, confirming that even otherwise healthy children may suffer from severe COVID-19 [9]. Finally, it has been definitively established that children, including neonates, can suffer from long-term effects of symptomatic and asymptomatic SARS-CoV-2 infection and develop multisystem inflammatory syndrome (MIS-C for children [10,11] and MIS-N for neonate [12,13]) and long-COVID (LC) [14,15,16]. MIS-C and MIS-N are rare conditions that develop 2–6 weeks after SARS-CoV-2 infection and can lead to hospitalization and death; LC that emerges after 3 months from acute SARS-CoV-2 infection is very common and can be detected in about 25% of pediatric COVID-19 cases with various clinical manifestations and whose duration and long-term effects are not precisely defined [17]. However, MIS-C/MIS-N for the severity and LC of frequency are clinically relevant and deserve attention for several aspects, including anti-COVID-19 specific therapy [18].

The increase in the incidence and severity of reported pediatric COVID-19 cases and the risk of long-term consequences seem to indicate that effective preventive and therapeutic measures, specifically prepared for use in children, are urgently needed. The development of effective vaccines for children has been too slow. As previously reported [6], only on 17 June 2022 mRNA vaccines for 6 months to 4-year-old children were authorized for emergency use in the USA. In the European Union, this occurred at the end of October 2022 [19]. The protection of infants < 6 months of age can be ensured only by vaccinating pregnant women [20], a practice that is not universally accepted, leaving unprotected several neonates and infants in the first months of life [21]. Furthermore, the planning of pediatric vaccination was suboptimal with the result that the vaccination coverage of children, especially the youngest, was generally very low [22]. Even less advanced is the development and testing of antivirals for the treatment of pediatric COVID-19. This means that contrarily to adults, several children at risk of or with SARS-CoV-2 infection cannot receive potentially useful pharmacologic measures. The main aim of this paper is to discuss which types of treatment are presently available for pediatric patients with COVID-19, which of them are authorized for pediatric age, and which are the most important limitations of COVID-19 therapy in neonates and children.

## 2. Anti-SARS-CoV-2 Measures Presently Authorized

Four different antiviral measures, remdesivir (RVD), the combination nirmatrelvir plus ritonavir (Paxlovid), molnupiravir (MPV), and the monoclonal antibody bebtelovimab (BEB), are presently approved or authorized for the emergency use of COVID-19 treatment by most national health authorities, although with limitations according to the clinical relevance of disease and the patient’s characteristics. The characteristics of each of these antivirals and what is known about each of them regarding their potential use in children are reported in the following sections.

### 2.1. Remdesivir

**Figure 1:**
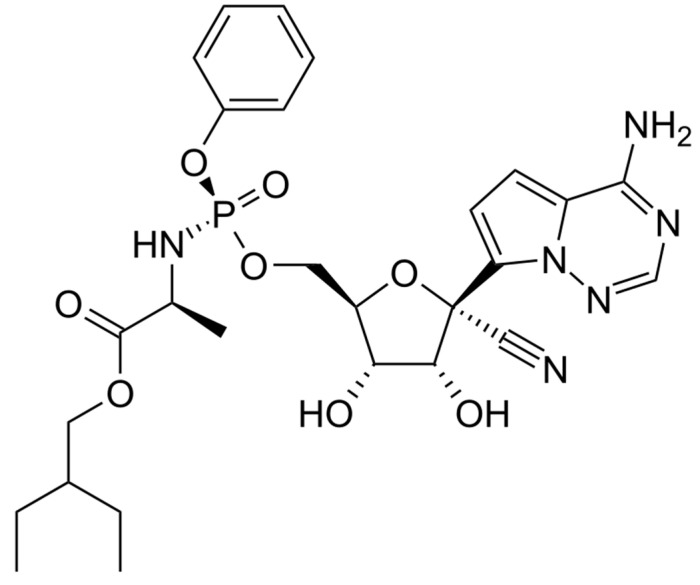
Remdesivir structure.

With the exception of neonates, RDV can be administered to patients of any age by an intravenous route. It is a phosphoramidite prodrug of a monophosphate nucleoside analog (GS-441524) that acts as a viral RNA-dependent RNA polymerase inhibitor (Figure 1).

Through this mechanism, it impairs the genome replication process of all coronaviruses, including SARS-CoV-2, and is able to become effective in the treatment of COVID-19 [23]. RDV emergency use in hospitalized pediatric patients with severe COVID-19 has been authorized by FDA since the first months of the pandemic. After several regulatory modifications, mainly including detailed indications for use in older children and adolescents, on 25 April 2022, FDA expanded the approval of this drug, including all children aged ≥ 28 days and weighing at least 3 kg. Moreover, the characteristics of the pediatric patients who could receive RDV were detailed. In particular, it was defined that RDV could be given to: (1) children with the above-mentioned characteristics of age and weight who were positive for a direct SARS-CoV-2 viral testing and were hospitalized for severe disease and (2) children maintained at home because of mild to moderate disease but with high risk for the progression of severe COVID-19, including hospitalization and death [24]. The recommended dosages for age and child characteristics are detailed in (Table 1).

Therapy may be extended up to 10 days if the patient does not clinically improve or requires extracorporeal membrane oxygenation or mechanical ventilation. Significant drug interactions exist, requiring dose/frequency adjustment or avoidance. Dosage adjustment is needed in the presence of renal insufficiency.

The availability of the pediatric use of a drug that was proved effective and is generally quite well tolerated and safe in adults can only be seen as a great advantage, especially for the youngest children for whom other potentially effective therapeutic measures are presently lacking or are in early development with use limited to mild to moderate cases. However, when studies used to support authorization are analyzed, it emerges that the procedures followed by experts to approve RDV use in children are totally different from those generally requested to license pediatric drugs. Most of the conclusions regarding the dosage, efficacy, safety, and tolerability of RDV in pediatrics were not derived from studies carried out in children but were simply extrapolated from studies performed in adults. The approval of RVD administration in patients aged ≥ 12 years and weighing ≥ 40 kg was given without any evaluation of the drug pharmacokinetics, taking into account only the data collected in adults. The knowledge that the absorption, distribution, metabolism, and elimination of drugs are generally similar in adolescents and adults was considered sufficient to give these children the same dosages that have proven effective and safe in adults. This conclusion was further strengthened by the evidence that using a mathematical model to incorporate age-dependent physiology and drug physicochemical properties to calculate the dosage used in adults could induce the children of this age group’s RVD blood levels and excretion of RVD metabolites substantially similar to those detected in adults [25]. Similar limitations can be found when the evaluation of the safety and tolerability of RVD in children aged ≥ 12 years and weighing ≥ 40 kg are considered. Despite no randomized controlled trial directly evaluating these variables in patients of this age and weight, the dosages recommended in adults were considered safe and well tolerated as, in studies enrolling adults, the incidence of adverse events was similar regardless of weight, including patients with weight significantly lower than 40 kg, i.e., in the range of most children ≤ 12 years of age. On the other hand, supporting the idea that RDV could be used in older children with a schedule similar to those recommended in adults is provided by the results of some studies in which children with these characteristics received RVD for compassionate use. In all the cases, a relatively low incidence of severe adverse events was evidenced [26,27]. Without direct support, this is also the conclusion regarding efficacy. Even in this case, data regarding efficacy in hospitalized children with severe disease are deduced from the cumulative evaluation of three clinical trials enrolling several hundred patients with COVID-19 receiving RDV. The studies showed that the drug was superior to the placebo in reducing the time to recovery [28]; treatment with 5-day and 10-day regimens was equally effective [29], and a 5-day treatment was more effective than standard treatment [30]. Unfortunately, all the patients, except one, were adults. Despite this, RDV was considered effective in older children and adolescents. Extrapolation was considered acceptable due to the similarity of COVID-19 clinical manifestations in adolescents and adults and the supposition that the response to an antiviral drug could not be different among these subjects [31]. Without any direct support, this is also the conclusion regarding the use of RDV in non-hospitalized children ≥ 12 years. In this case, rules for RDV use were extrapolated by the results of a study in which it was found that a 3-day treatment of adults at high risk of disease progression was associated with a significant 87% reduction in the risk of COVID-19-related hospitalization or all-cause death by Day 28 (0.7% vs. 5.3%, *p* = 0.008) [32].

Even greater limitations can be found in studies performed to authorize RDV administration in children < 12 years, particularly infants and younger children. Pharmacokinetic data, leading to the definition of RDV dosages in this age group, were collected in the Sponsor’s Initial Pediatric Study Plan that initially enrolled only 53 children, among whom very few were infants and toddlers, and many were school-age children leading to median age of the studied patients of 7 years [33]. Lacking data directly collected in infants and younger children, the RDV used on these patients was authorized simply considering the results of a complex calculation: through a mathematical model, it was concluded that the doses recommended to children could assure RDV exposures would not be different from those deemed safe and effective in adults, justifying not only the pediatric dosage but also the supposition that in younger children safety, tolerability, and clinical efficacy could be quite similar to those found in adults [33]. Particularly in very young infants, these conclusions, especially those concerning safety and tolerability, remain debatable, and the use of RDV in these subjects should be evaluated with extreme caution. The administration of RDV is not free from the development of adverse events. In the Sponsor’s Initial Pediatric Study Plan [33], the most common adverse event in patients taking RDV was constipation (17%), followed by acute kidney injury (11%), hyperglycemia (9%), and pyrexia (9%). Additionally, 8% of participants had an increase in alanine transaminase. Renal function is immature at birth, even in term infants. Maturations develop slowly, and in most children, an adult function is reached after the end of the first year of age (later in preterm infants) [34]. RDV and its active metabolite are predominantly (74%) and renally eliminated. This means that in younger children, RDV and its metabolites can accumulate and cause an increase in adverse events. Moreover, drug accumulation and the increase in adverse event incidence can be favored by direct renal damage due to RDV or renal insufficiency due to COVID-19 itself [35]. It is very likely that the acceleration in the authorization processes for the pediatric use of RDV has been depended on due to the need to tackle the very serious problems posed by the COVID-19 pandemic. However, it seems necessary that, given the increase in cases described in younger children, ad hoc studies for a more precise evaluation of the effective dosage and safety and tolerability of the drug were carried out. The inclusion of the ongoing Sponsor’s Initial Pediatric Study Plan of two cohorts of newborns of different gestational ages could offer some further useful information in this regard [33].

### 2.2. Nirmatrelvir plus Ritonavir

**Figure 2:**
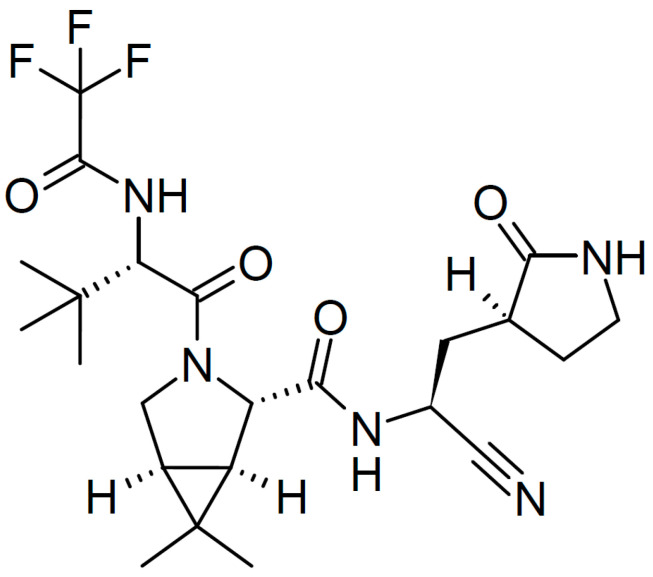
Structure of nirmatrelvir.

On 22 December 2021, the FDA issued an emergency use authorization for the association nirmatrelvir plus ritonavir (Paxlovid, PV) for the treatment of mild-to-moderate COVID-19 in adults and pediatric patients (12 years of age and older weighing at least 40 kg) with positive results of direct SARS-CoV-2 viral testing, and who were at high risk for progression to severe COVID-19, including hospitalization and death [36]. Figure 2 shows the structure of nirmatrelvir.

The recommended dosages were 300 mg nirmatrelvir and 100 mg ritonavir two times a day for 5 days. Treatment should be initiated as soon as possible and within 5 days of symptom onset. Moreover, it was highlighted that the use of PV could be associated with the development of serious adverse reactions due to the interaction of ritonavir with other drugs [36].

Nirmatrelvir is an oral drug that inhibits the SARS-CoV-2 main protease, therefore, impairing viral replication [37]. It is associated with ritonavir, an HIV-1 protease inhibitor that is not active against SARS-CoV-2, due to the ability of this drug to increase nirmatrelvir bioavailability through the inhibition of CYP3A, a liver enzyme that metabolizes nirmatrelvir. Plasma concentrations of nirmatrelvir are maintained higher and longer than those able to inhibit SARS-CoV-2 replication. Unfortunately, CYP3A metabolizes several other drugs, and the plasma levels of these can be significantly increased during PV use. Moreover, when drugs influencing CYP3 activity are initiated, variations in PV activity can occur.

Authorization for use in adults was mainly based on the results of a study enrolling adults with mild to moderate SARS-CoV-2 infection and prespecified risk factors for progression to severe disease and subjects aged 60 years or more regardless of underlying chronic medical conditions [38]. A group of matched controls were given a placebo and also enrolled. All patients had not received a COVID-19 vaccine and had not been previously infected with SARS-CoV-2. Compared to the controls, PV administration within 5 days from infection was associated with a significant reduction (88%) in the risk of hospitalization and death from any cause. Only 0.8% of patients who received PV were hospitalized or died during 28 days of follow-up compared to 6% of the patients who received the placebo. The viral load was lower with PV than with the placebo at day 5 of treatment, with an adjusted mean difference of −0.868 log10 copies/mL when treatment was initiated within 3 days after the onset of symptoms. The safety and tolerability of the combination were apparently very good, as the incidence of adverse events developed during the study period was similar in the two groups (22.6% with PV and 23.9% with placebo) [38]. However, serious adverse events and adverse events leading to treatment discontinuation were significantly less common among the treated children than among the controls (1.6% vs. 6.6% and 2.1% vs. 4.2%, respectively). Dysgeusia (5.6% vs. 0.3%) and diarrhea (3.1% vs. 1.6%) occurred more frequently with nirmatrelvir plus ritonavir than with the placebo.

Studies were carried out after FDA authorization had confirmed the efficacy and safety of PV in adults. A meta-analysis of 13 studies involving 186,306 patients showed that the overall odds ratio (OR) for death and hospitalization among COVID-19 patients receiving PV compared to the placebo group was 0.22 (*p* < 0.0001) [39]. Separate evaluations of the risk of hospitalization and mortality revealed a reduction of 68% and 88%, respectively. The risk of virologic rebound some days after treatment cessation, described by several reports, was not documented. The combined analysis of three studies in this regard showed that the overall risk of rebound was similar in patients receiving PV and the controls (OR 0.99, *p* = 0.99) [40,41,42].

Regarding children, data were totally lacking. The pharmacokinetics of nirmatrelvir in pediatrics was never evaluated. Moreover, randomized controlled trials studying the efficacy, safety, and tolerability of PV in children were never carried out. Only uncontrolled experiences of PV administration in children aged 6–14 years showing that the combination could be a feasible option to treat SARS-CoV-2-infected children have been reported [43]. It seems likely that the authorization for PV use in patients ≥ 12 years old weighing ≥ 40 kg was decided only based on the supposition that older children and adolescents have similar COVID-19 and similar physiological characteristics, including drug metabolism. Nothing is known about the administration of PV in children < 7 years of age, particularly in the youngest. The pharmacokinetics of drugs, mainly metabolism and excretion, are different in younger children than in adults [44]. The inhibition of liver enzymes such as CYP3A by ritonavir and the effect on nirmatrelvir level can vary significantly according to age. Moreover, children with underlying diseases that are potentially the most important target of PV use generally receive drugs that are metabolized by CYP3A. Anticonvulsant agents (phenobarbital, phenytoin, carbamazepine, clonazepam) and systemic corticosteroids (betamethasone, dexamethasone), antibiotics (erythromycin, rifabutin, rifampin), are the most common among those more frequently used in pediatrics. The introduction of PV may lead to relevant modifications of their efficacy due to the increase or decrease in nirmatrelvir plasma concentrations and the risk of an adverse event or poor treatment efficacy, respectively [45].

### 2.3. Bebtelovimab

Bebtelovimab (BEB) is the only anti-SARS-CoV-2 monoclonal antibody that is in vitro active against the current dominant circulating Omicron subvariants [46]. It is authorized by FDA for the emergency use in mild-to-moderate COVID-19 adults and pediatric patients (12 years of age and older weighing at least 40 kg) with positive results of direct SARS-CoV-2 viral testing in those who are at high risk of progression to severe COVID-19, including hospitalization or death, and for whom alternative COVID-19 treatment options approved authorized by the FDA are not accessible or clinically appropriate [47]. The recommended dose of 175 mg as a single IV injection was selected using a quantitative modeling and simulation framework in order to identify a dose resulting in a drug concentration inhibiting virus replication in ≥90% of patients for ≥28 days [48]. BEB should be administered as soon as possible after infection detection and within 7 days of symptom onset [47]. Authorization was decided, although no clinical data had established the efficacy of BEB for the treatment of COVID-19 caused by the SARS-CoV-2 Omicron variant. In this regard, it was considered sufficient that this pharmacological measure acted with a mechanism of action quite similar to other anti-SARS-CoV-2 monoclonal antibodies that were previously found to be effective in reducing hospitalization and death rates in hospitalized high-risk patients [49]. Moreover, further support for the authorization was given by a study carried out before the emergence of the Omicron variant. In this study, adults (mean age 35 years) with mild to moderate COVID-19 at low risk of negative evolution were randomly assigned to receive a placebo, BEB, or BEB together with two other monoclonal antibodies, bamlanivimab (700 mg) and etesevimab (1400 mg) [50]. By day 7 after IV injection, a persistent high viral load was detected in a lower number of patients receiving the placebo (19.8%) than in those receiving the combined monoclonal antibody therapy (12.7%) or BBE (12.0%). Differences between the groups were not statistically significant. By day 11, differences in the viral load became significant for both BEB and the combined therapy (*p* = 0.006 and 0.043, respectively). The treated patients had a more rapid symptoms resolution (median 2 days for patients treated with BEB and 1 day for patients treated with the multiple therapies) compared to the placebo (8 days). Hospitalization rates and all-cause deaths by day 29 were similar in all the study groups [50]. Data regarding children were not collected.

As for remdesivir and PV, the authorization of BEE administration in children 12 years of age and older weighing at least 40 kg was decided by extrapolating data collected from adults. Additionally, in this case, the similarity of COVID-19 manifestations and response to drug administration in adolescents and adults allowed for extrapolation. However, before the use of BEB in younger children, an adequate evaluation of dosages should be performed.

### 2.4. Molnupiravir

**Figure 3:**
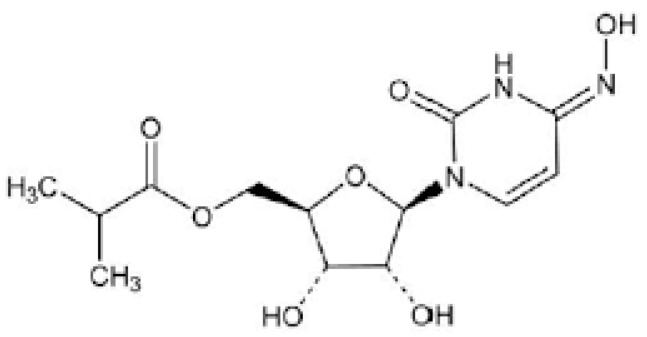
Structure of molnupiravir.

MPV is a prodrug that is metabolized to the ribonucleoside analog N-hydroxycytidine (NHC), which is phosphorylated to form the pharmacologically active ribonucleoside triphosphate (NHC-TP). Figure 3 shows the MPV structure.

NHC-TP incorporation into SARS-CoV-2 RNA by the viral RNA polymerase results in an accumulation of errors in the viral genome, leading to the inhibition of replication and a potentially significant effect in COVID-19 treatment [51]. MPV is authorized for emergency use for the treatment of mild-to-moderate coronavirus disease 2019 (COVID-19) in adults who are at high risk of progression to severe COVID-19, including hospitalization or death, when alternative COVID-19 treatment options by the FDA are not accessible or clinically appropriate [52]. The recommended dosage is 800 mg two times a day for 5 days, given as soon as possible in the first 5 days after infection. Contrary to other presently available anti-SARS-CoV-2 agents, MPV is not authorized for use in children because it may affect bone and cartilage growth. Studies carried out in experimental animals have shown that long-term high-dose administration of MPV could cause an increased thickness of the growth cartilage of the epiphysis of long bones and patella associated with decreased osteogenesis and decreased trabecular bone in the metaphysis [53]. Moreover, due to its mutagenic potential, it cannot be administered to pregnant women.

The emergency use authorization was decided based on the results of a study in which drug administration was associated with a 30% reduction in the risk of hospitalization and death in treated patients compared to the controls (6.8% vs. 9.7%) [54]. Tolerability and safety were found to be good in the study during treatment, and 14 days after the last dose was given, less than 10% of patients showed mild to moderate adverse events such as diarrhea (3%), nausea (2%), dizziness (1%), and headache (1%). No serious adverse event was reported. However, due to the risk of bone damage, use in children cannot be hypnotized.

## 3. Conclusions

Approved antiviral drugs for use in children with COVID-19 are still extremely limited. Some of the drugs presently authorized for emergency use in adults, such as MPV, cannot be used in children for the risk of adverse events regarding bone growth. The other antivirals can be used, at least in older children, and RDV in all children except in neonates. However, lacking pediatric pharmacokinetic studies and randomized, controlled clinical trials, it is not certain that the use of these drugs, according to official recommendations, is really effective and safe. Throughout the first phase of the pandemic, the evidence that COVID-19 was generally less frequent and severe in children than in adults strongly conditioned studies to aim instead at verifying the effective efficacy as well as the safety and tolerability in the different pediatric ages of antivirals evaluated for use in adults. Probably due to the different epidemiological and clinical characteristics of pediatric COVID-19 compared to adults, it seems likely that pediatric research was considered not indispensable and difficult to perform, mainly because the number of children enrolled in each study to obtain beneficial results was very high. The decision to extrapolate the tolerability, safety, and efficacy of each antiviral from results carried out in adults probably arose from these considerations. It seems likely that with the increase in the number of severe cases of pediatric COVID-19, a wider use of RDV for the therapy of hospitalized children is being made. In this regard, careful verification of the kinetics of the drug in the first stages of life and its effective efficacy in controlled clinical studies seems essential. Moreover, special attention should be paid to the use of Paxlovid and BEB. If extrapolation of data collected in adults seems rational for adolescents ≥ 12 years of age, the administration of these antivirals to younger children for compassionate use cannot be recommended until careful research on pharmacokinetic and clinical data specifically collected from these patients are made available.

## Figures and Tables

**Table 1:** Recommended dosage of remdesivir in neonates and children.

Dosages According to Age and Weight
Neonates weighing < 3.5 kg: Loading dose 2.5 to 5 mg/kg IV on day 1, followed by 1.25 mg/kg/dose IV once daily on day 2–5. Neonates weighing ≥ 3.5 kg: Loading dose 5 mg/kg IV on day 1, followed by 2.5 mg/kg/dose IV once daily on day 2–5. 3 to <40 kg: Loading dose: 5 mg/kg/dose IV on day 1, followed by 2.5 mg/kg/dose IV once daily on day 2–5. ≥40 kg: Loading dose: 200 mg IV on day 1, followed by 100 mg IV once daily on day 2–5.

## Data Availability

Data sharing not applicable.
